# Living Up to a Name: Gender Role Behavior Varies With Forename Gender Typicality

**DOI:** 10.3389/fpsyg.2020.604848

**Published:** 2021-01-22

**Authors:** Gerianne M. Alexander, Kendall John, Tracy Hammond, Joanna Lahey

**Affiliations:** ^1^Department of Psychological and Brain Sciences, Texas A&M University, College Station, TX, United States; ^2^Department of Computer Science and Engineering, Texas A&M University, College Station, TX, United States; ^3^Bush School of Government and Public Service, Texas A&M University, College Station, TX, United States

**Keywords:** forenames, gender, forename stereotypes, gender stereotypes, gender differences

## Abstract

Forenames serve as proxies for gender labels that activate gender stereotypes and gender socialization. Unlike rigid binary gender categories, they differ in the degree to which they are perceived as “masculine” or “feminine.” We examined the novel hypothesis that the ability of a forename to signal gender is associated with gender role behavior in women (*n* = 215) and men (*n* = 127; *M* = 19.32, *SD* = 2.11) as part of a larger study evaluating forenames used in resume research. Compared to individuals endorsing a “gender-strong” forename, those perceiving their forename as relatively “gender-weak” reported less gender-typical childhood social behavior and a weaker expression of gender-linked personality traits. Our findings suggest that forenames strengthen or weaken gender socialization, gender identification, and so contribute to the variable expression of gender role behavior within binary gender groups.

## Introduction

A binary gender label at birth is a cultural proxy for the male or female appearance of external genitalia, a physical trait that usually corresponds to the sexual differentiation of brain structure and function proposed to support a male-typical or female-typical behavioral phenotype (Berenbaum and Beltz, 2016). According to established theories of gender development, a body-based gender label also activates a process of gender socialization whereby behavior is shaped to conform to the expected social roles for women and men (Wood and Eagly, 2002) through the general learning principles of modeling, reinforcement, and punishment (Bussey and Bandura, 1999). Cognitive development in early childhood supports gender-typing further by allowing the internalization of a gender label and the elaboration of a gender schema, a cognitive network of associations between beliefs, interests, and activities prescribed to that gender by society (Bem, 1981; Martin and Halverson, 1981). By directing attention to gender-relevant stimuli throughout development, gender schemas also result in the selective encoding of information that ultimately defines the gendered-self and sustains the expression of gender-linked behavior even in the absence of external factors (Martin et al., 2002).

Biological and cognitive-social theories propose similar behavioral phenotypes based on the binary gender group assignment at birth (e.g., males will be more aggressive, whereas females will be more sensitive). Yet, although infants may be born prepared to be gendered (Alexander, 2003), gender differences in human behavior are generally small in magnitude, with exceptions being noted for some personality traits (e.g., sensation seeking and agreeableness), cognitive abilities (3D mental rotation), and social interests (people vs. things; for review, see Hyde, 2014). Further, a general finding across studies is that any behavioral differences between binary gender groups are typically smaller than the within-in group variability (Maccoby, 1988; Ellemers, 2018).

A variety of factors are proposed to explain the highly variable expression of gender-linked behavior within binary gender groups, including biologically based behavioral tendencies, parental attitudes towards gender, and the sex composition of the family (Endendijk et al., 2018). Additionally, because genitalia are typically hidden from the view of others in society, gender socialization is often dependent on culturally defined cues to communicate an individual’s gender group assignment. Research on generalization indicates response to such cues is typically weaker than response to the primary stimulus (Ghirlanda and Enquist, 2003), suggesting that these less robust gender signals are another source of variability in gender socialization processes that influence developmental outcomes. For example, some gender cues, like clothing, colors, and playthings (Pomerleau et al., 1990), are age and context dependent and vary in signal strength as a function of differences in gendered parenting practices and beliefs (Mesman and Groeneveld, 2018). It may be argued that forenames resolve any resulting ambiguity in gender categorization caused by less robust gender cues, as forenames are typically consistent across the lifespan and are used from early childhood to effectively categorize individuals as male or female (Alford, 1988; Bauer and Coyne, 1997; Gelman et al., 2004). However, although gender group assignment can be reliably inferred on the basis of a forename, the information forenames convey about gender is on a continuum. On one hand, forenames are clearly connected to a gender binary system (Robnett, 2017), but unlike a binary gender label they signal varying levels of behavioral traits within a gender group (Kasof, 1993; Newman et al., 2018), with some gender-specific forenames being viewed as more prototypical than others (Van Fleet and Atwater, 1997).

Like other stereotypes, forename stereotypes, which include associations to race and ethnicity (Kasof, 1993; Barlow and Lahey, 2018), are viewed as self-fulfilling prophesies realized through the differential expectations and treatment of individuals by others in society (Erwin, 1995). From that perspective, forenames chosen at birth may represent an efficient means by which parents communicate expectations and beliefs about gender to others, which then become internalized in adolescents and emerging adults (Epstein and Ward, 2011). Peers are likely another important factor in the realization of forename stereotypes, consistent with the early use of forenames to infer gender (Bauer and Coyne, 1997; Gelman et al., 2004) and the sanctioning of counter stereotypical behavior, first around toy play in early childhood (Skočajić et al., 2020) and then more broadly in adolescence (Brechwald and Prinstein, 2011).

Previous researchers have suggested that the perception of a forename as “gender-wrong” (e.g., a boy named Sue or a girl named Mark) by the self or by others can disrupt developing feminine or masculine identities (Pilcher, 2017), self-esteem or adjustment (Ellington et al., 1980; Figlio, 2007). If so, then the prototypical strength of a gender-specific forename may similarly, but likely more subtly, shape the development of the gendered self. Early tests of a relationship between forenames and gender-linked behavior considered differences between gender-typical forenames and the less commonly assigned “androgynous” or “ambiguous” forename with inconsistent results (e.g., Rickel and Anderson, 1981; Mehrabian, 2001). However, compared to gender-typical forenames, androgynous forenames are rarely encountered and differ in other evaluative dimensions of personality (Mehrabian, 2001). Therefore, whether the perception of a gender-linked forename as “gender-weak” is associated with corresponding changes in gender-typed behavior is not yet known.

The present study addresses a call for greater research on naming practices, including gender-typed forenames (Robnett, 2017), by measuring the association between the self-perception of forenames and two domains of gender-linked behavior: social play and personality. Adult’s retrospective reports of childhood play are sensitive to other factors proposed to contribute to within-sex variability in gender-linked behavior, namely prenatal androgen exposure (Hines et al., 2004), suggesting these recalled behaviors may also be sensitive to any forename effects on gender development. We also included a measure of gender-linked personality thought to measure the internalization of cultural norms for gendered behavior and used in early research on androgynous forenames (Rickel and Anderson, 1981). Based on theory outlined in Pilcher (2017), we hypothesized that the degree to which an individual believes their forename is gender-typical would be positively associated with levels of recalled social play and the strength of gender-linked personality traits within gender groups.

## Materials and Methods

The sample included women (*n* = 215) and men (*n* = 127) ranging in age from 18 years to 45 years (*M* = 19.32, *SD* = 2.11), recruited from an undergraduate psychology subject pool at a large Southwestern University in Fall 2018-Summer 2019 as part of ongoing resume research approved by the institutional review board overseeing human subject research. Participants completed an online Qualtrics survey where they rated the perceived masculinity and femininity of their forename and their self on scales from 0 (not at all) to 100 (extremely) and completed two questionnaire measures of gender-linked behavior. Race and ethnicity were measured independently. The majority of the participants identified their race as White (82.7%), followed by Asian (9.6%) and Black or African-American (2.9%). Of these, 28% identified themselves identify as Hispanic or Latino (See Table 1 for complete demographics). Excluded from this final sample were seven individuals who did not respond to the gender question (Do you identify as male, female or other?), four individuals who selected “other” in response to the gender question, and two individuals that did not complete the gender-role questionnaires.

**Table 1:** Demographics.

	Male	Female	Total
Age M (SD)	19.21 (1.55)	19.38 (2.36)	19.32 (2.11)
Race (%)			
White	84.9%	82.4%	82.7%
Black	2.4%	2.8%	2.9%
Asian	9.5%	9.7%	9.6%
American Native/Indian	0	1.4%	1.2%
Native Hawaiian/pacific islander	0	0.9%	0.6%
Did not respond	2.4%	2.8%	2.9%
Ethnicity (%)			
Hispanic or Latino	23.8%	27.0%	25.8%
Not Hispanic or Latino	76.2%	73.0%	74.2%

Race and Ethnicity were measured independently. Race was measured using a multiple choice question that asked participants to identify which one race of the five options listed they identified with or to indicate that they would prefer not to respond. Ethnicity was measured using the item “Do you identify as Hispanic or Latino?” to which participants indicated “yes” or “no.”

*Recalled childhood gender-role behavior* was measured by the 24-item Pre-School Activities Inventory (PSAI) that asks adults to recall the frequency of engaging in a variety gender-typical play activities (e.g., playing with toys such as dolls and trains, engaging in activities such as climbing, fighting, or playing house). Higher scores indicate higher frequencies of male-typical activities and lower scores indicate higher frequencies of female-typical activities. The measure has good reliability in children (test-retest reliability = 0.65; split half reliability = 0.88; Golombok and Rust, 1993). When used with adults, the scores show very large gender differences (Cohen’s *d* = 2.65–3.25; Hines et al., 2004; Alexander, 2006; Alexander and Evardone, 2008) and are positively correlated with an implicit measure of adult visual interest on gender-linked toys and activities (i.e., eye-tracking measures of visual fixations and looking times; Alexander, 2006). In this sample, the PSAI showed good internal consistency within the female-typical items (Cronbach’s *α* = 0.93) and male-typical items (Cronbach’s *α* = 0.89).

Personality dimensions of *adult gender-role behavior* were assessed by the 60-item Bem Sex-Role Inventory (BSRI), a widely used scale with good (*r* > 0.80) test-rest reliability and shows stability in gender differences across time periods (Bem, 1974; Donnelly and Twenge, 2017). Ratings of personality traits on a 7-point scale averaged across 20 masculine (e.g., competitive, ambitious, and independent) and 20 feminine characteristics (e.g., cheerful, understanding, and tender) yield measures of masculine and feminine personality dimensions, with higher scores on each scale indicating stronger expression of those traits. There is good internal consistency across both the masculine (Cronbach’s *α* = 0.88) and feminine subscales (Cronbach’s *α* = 0.85). A median split of scores on the two scales is used to differentiate individuals who are sex-typed (e.g., women scoring above the median on the feminine scale and below the median on the masculine scale), cross sex-typed (e.g., women scoring below the median on the feminine scale and above the median on the masculine scale), androgynous (i.e., individuals scoring above the median on both masculine and feminine scales), or undifferentiated (i.e., individuals scoring below the median on both masculine and feminine scales; Bem, 1981).

### Statistical Analyses

**Figure 1:**
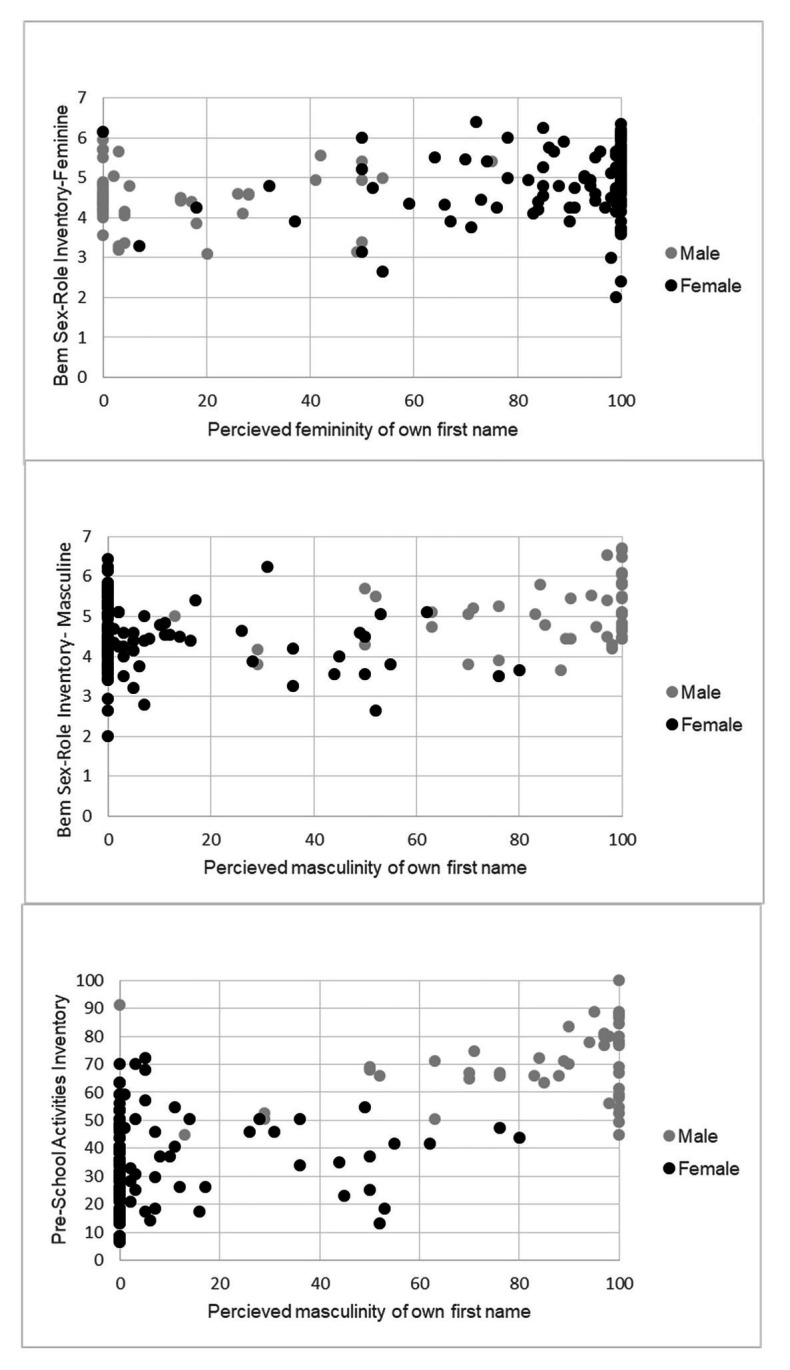
Self-perception of name and gendered behavior.

As illustrated in Figure 1, forename ratings were skewed towards extreme assessments of masculinity and femininity. Therefore, in addition to an exploratory analyses of relationships using correlational analyses, we used Multivariate ANOVA (MANOVA) to compare behavior in individuals describing their forename as prototypical (i.e., 100% gender-typed) and those describing their forename as less than prototypical (i.e., less than 100% gender-typed). Finally, similar to the methods reported in earlier research (Rickel and Anderson, 1981), we used a Chi-Square test to examine the relationship between these two categories of forename gender-typing and the BSRI classification of participants as sex-typed, cross-sex-typed, undifferentiated, and androgynous. To allow interpretation of any group differences, we also report the most widely used measure of gender differences (Hyde, 2014), Cohen’s *d* (Cohen, 1988), where values of 0.20, 0.50, and 0.80 indicate small, moderate, and large effects, respectively.

## Results

For men, the perceived masculinity of their forename was strongly associated with the perceived masculinity of the self, *r*(127) = 0.69, *p* < 0.001. The relationship between women’s perceptions of the femininity of their forename and the self was also positive, *r*(215) = 0.29, *p* < 0.001, but the association was weaker than that observed in men, Fisher’s *z* = 4.86, *p* < 0.001. Figure 1 illustrates the associations between forename name ratings of masculinity and femininity and questionnaire measures of gender role behavior. Higher femininity ratings of forenames were associated with higher scores on the BSRI-Fem, *r*(342) = 0.25, *p* < 0.001, and lower (i.e., less masculine) scores on the PSAI, *r*(324) = −0.73, *p* < 0.001. Higher masculinity ratings of forenames were associated with higher scores on the BSRI-Masc, *r*(342) = 0.34, *p* < 0.001 and higher (i.e., more masculine) scores on the PSAI, *r*(324) = 0.73, *p* < 0.001.

Table 2 summarizes the scores on childhood and adult gender-role behavior measures in the four groups defined by ratings of forenames as 100% gender-typed or less than 100% gender-typed. As expected, the ratings of forename masculinity in men rating their name 100% gender-typed (*n* = 52) vs. men rating their name less than 100% gender-typed (*n* = 75) showed large differences (*M* = 100.00, *SD* = 0.0 vs. *M* = 70.71, *SD* = 22.23, *d* = 1.86). In addition, forename femininity ratings were markedly higher in men in the weaker gender-typed forename subgroup compared to men in the stronger gender-typed forename subgroup (*M* = 20.88, *SD* = 20.28 vs. *M* = 1.86, *SD* = 5.89, *d* = 1.86). Similarly, the ratings of forename femininity in women rating their name 100% gender-typed (*n* = 92) vs. women rating their name less than 100% gender-typed (*n* = 123) showed large differences in forename femininity ratings (*M* = 100, *SD* = 0.0 vs. *M* = 80.65, *SD* = 21.50, *d* = 1.27). Forename masculinity ratings were higher in women in the weaker gender-typed forename subgroup compared to women in the stronger gender-typed forename subgroup (*M* = 15.78, *SD* = 22.20 vs. *M* = 1.98, *SD* = 11.22, *d* = 0.78).

**Table 2:** Gendered measures across men and women with strong or weak gender identifying names.

	Men (*n* = 127)		Women (*n* = 215)		Total (*n* = 342)	
	Strong name	Weak name	Strong name	Weak name	Strong name	Weak name
	M (SD)	M (SD)	M (SD)	M (SD)	M (SD)	M (SD)
Bem sex-role inventory						
Masculine	5.37 (0.78)	4.96 (0.70)	4.63 (0.78)	4.48 (0.84)	4.89 (0.85)	4.66 (0.82)
Feminine	4.62 (0.70)	4.52 (0.74)	5.10 (0.81)	4.73 (0.77)	4.93 (0.80)	4.65 (0.76)
Pre-school activities inventory	73.00 (12.84)	69.68 (11.33)	31.87 (15.51)	37.27 (15.03)	46.72 (24.60)	49.54 (20.89)

Bem Sex-Role Inventory scores are the average response to 20 items per subscale scored from 1 (never or almost never true) to 7 (almost always true). The Pre-School Activities Inventory is scored so higher values reflect more masculine-typical behavior.

Multivariate ANOVA using Forename Strength (100% or stronger gender-typed vs. less than 100% or weaker gender-typed) and Gender (male vs. female) as grouping factors on gender-role behavior measures (i.e., BSRI-M, BSRI-F, and PSAI scores) showed a main effect of Gender, Multivariate *F*(3,336) = 178.79, *p* < 0.001, a main effect of Forename Strength, Multivariate *F*(3,336) = 5.01, *p* = 0.002, and a Gender by Forename Strength interaction, Multivariate *F*(3,336) = 3.39, *p* = 0.018. The Univariate *F*’s showed the expected main effects of Gender on scores on the BSRI-M, *F*(1,338) = 46.71, *p* < 0.001, BSRI-F, *F*(1,338) = 15.62, *p* < 0.001, and PSAI, *F*(1,338) = 526.25, *p* < 0.001. Consistent with gender differences on these measures, men compared to women reported more masculine and less feminine personality traits on the BSRI and reported engaging in higher frequencies of male-typical social behavior during childhood on the PSAI. A Gender by Forename Strength of forename interaction on PSAI scores was significant, *F*(1,338) = 7.40, *p* = 0.007. Men in the strong gender-typed forename group compared to men in the weaker gender-typed forename group reported higher (i.e., more male-typical) scores on the PSAI (*d* = 0.27). The opposite pattern occurred in women: women in the strong gender-typed forename group compared to women in the weaker gender-typed forename group reported lower (i.e., more female-typical) scores on the PSAI (*d* = 0.35). Finally, there was an overall Gender-Strength of forename group effect on BSRI-M, *F*(1,338) = 9.84, *p* = 0.002, and BSRI-F, *F*(1,338) = 7.43, *p* = 0.007. Men and women in the stronger gender-typed forename groups reported higher BSRI-M scores (*d* = 0.57 for men and *d* = 0.17 for women) and higher BSRI-F scores compared to their counterparts in the weaker gender-typed forename group (*d* = 0.47 for women and *d* = 0.17 for men). The between-gender group differences on these two scales was stronger in the group of women and men reporting strong gender-typed forenames (*d* = 0.97 for BSRI-M and *d* = 0.62 for BSRI-M) compared to the gender group difference in individuals reporting weaker gender-typed forenames (*d* = 0.60 for BSRI-M and *d* = 0.27 for BSRI-F). However, the interaction between Gender and Forename Strength did not reach significance for either the BSRI-M, *F*(1,338) = 2.33, *p* = 0.127, or the BSRI-F, *F*(1,338) = 2.41, *p* = 0.121.

A 2 (Forename Strength) X 4 (Bem Categories) multidimensional contingency table showed a significant association between Forename Strength and Bem Categories (*x*^2^(3) = 12.35, *p* < 0.006). An examination of adjusted residuals greater than 1.96 (i.e., *p* < 0.05) showed that individuals reporting weaker gender-typed forenames compared to those reporting strong gender-typed forenames were more likely to be categorized as cross-sex-typed (15.66% vs. 6.94%) and less likely to be categorized as androgynous (23.23% vs. 35.11%), as shown in Table 3.

**Table 3:** Forename strength across differing Bem Sex-Role Inventory (BRSI) identity classifications.

	Sex-typed	Cross-sex-typed	Androgynous	Undifferentiated
Weak names				
Men *n*	36.00% 27	14.67% 11	26.67% 20	22.67% 17
Women *n*	29.26% 36	16.26% 20	21.14% 26	33.33% 41
Strong names				
Men *n*	38.46% 20	5.77% 3	38.46% 20	17.31% 9
Women *n*	33.70% 31	7.61% 7	34.78% 32	23.91% 22
Total				
Men *n*	37.00% 47	11.02% 14	31.50% 40	20.47% 26
Women *n*	31.16% 67	12.56% 27	26.98% 58	29.30% 63

Participants described their forename as either prototypical for their gender (100% gender-typed or gender strong) or less than prototypical (<100% gender-typed or gender weak).

## Discussion

Men and women in this research differed predictably in their perception of the masculinity and femininity of their forenames. However, consistent with findings that gender-specific forenames differ in their gender-typicality (Van Fleet and Atwater, 1997), a majority of men (59.1%) and women (57.2%) perceived their forenames as less than prototypically gender-typed. Compared to men endorsing a “gender-strong” forename, men perceiving their forename as relatively less masculine reported less male-typical childhood social behavior, as measured by the Preschool Activity Inventory (PSAI), and weaker masculine personality traits, as measured by the Bem Sex Role Inventory (BSRI). Similarly, compared to women endorsing a “gender-strong” forename, women perceiving their forename as less feminine reported higher levels of male-typical childhood social behavior and weaker feminine personality traits on these same measures. Across both gender groups, those rating their forenames as less prototypical were categorized more frequently as cross-sex-typed and categorized less frequently as androgynous on the BSRI. These associations across domains of childhood behavior and adult personality suggest that forename effects on gender-linked behavior may emerge in early childhood and continue into later adult life.

One explanation for our general results is that the variable signaling of masculinity and femininity by forenames influences the expression of gender-linked behavior through the differential treatment of individuals (Erwin, 1995; i.e., gender socialization). Findings from computer and behavioral science showing forename classifications of faces exceed chance suggest that even individual appearances (e.g., hairstyle and style of glasses) can be shaped to conform to forename stereotypes (Chen et al., 2013; Zwebner et al., 2017). However, external influences are less able to explain why letters in a forename predict letters in the names of residential locations and occupations of individuals (Pelham et al., 2002). Rather, this implicit valuing of forename perceptual features is proposed to occur because forenames become part of the self and generate positive attitudes about objects associated with that aspect of the self (“implicit egoism”).

An internalization of forenames with the gendered self is specifically addressed by the concept of “embodied named identity” (Pilcher, 2016), highlighting causal relations among body phenotypes, forename assignments, and the construction of identities including gender (Pilcher, 2017). The proposal that “our names are both constituted by and help to constitute our sexed and gendered selves” (Pilcher, 2016, p. 776) is consistent with the observed associations between ratings of self, forenames, and behavior in this research; however, establishing any causality will require additional investigation. Possible sources of evidence include studies showing that the gender typicality of forenames corresponds to the actual gender-specific treatment of children, the strength of children’s gender identification, or the associated elaboration of gender schemas in childhood that are proposed to sustain gender congruent behavior (Martin and Halverson, 1981). As facial appearances appear to conform to forename stereotypes (e.g., Chen et al., 2013), it is possible that the embodied gender-typicality of forenames also influences the degree to which body appearances conform to gender stereotypical ideals. Therefore, future research might also consider whether the gender-typicality of forenames influences the pursuit of body ideals (i.e., thinness in women and muscularity in men) thought to contribute to anorexia in women and muscle dysphoria in men (Griffiths and Yager, 2019) and explain a greater prevalence of eating disorders in transgender populations (Duffy et al., 2019).

Nearly 40 years ago, Maccoby (1988) proposed that the concepts of “masculinity” and “femininity” are fuzzy-sets, defined elsewhere as sets containing elements with varying degrees of membership that can be operationalized by any real value between 0 (fully out) and 1 (fully in; Pennings, 2003; Ragin and Pennings, 2005). Maccoby noted that one could be more or less masculine or feminine but not more or less male or female. However, consistent with the non-rigid boundaries of male and female identities (Diamond, 2020), gender identity is viewed more recently as a concept that can also be understood as a graded set of conditions (Ragin and Pennings, 2005). One proposal is that gender identity consists of four dimensions: gender typicality, felt pressure to conform, gender contentment, and intergroup bias (Egan and Perry, 2001), with gender typicality being positively associated with engagement in gender-typical activities consistent with our general findings for forename typicality. A body-based assignment of gender at birth and pursuit of gender confirming surgery in later life (Morrison et al., 2017) indicate body phenotypes are another condition of gender identity. Choosing a forename to embody gender identity is a common occurrence at birth and described as a fundamental aspect of a transgender individual’s transition (VanderSchans, 2015), consistent with our hypothesis that forenames influence the expression of gender-typed behavior because they strengthen or weaken gender identification (i.e., they are an additional condition in the set). It is clear that different pathways to gender identity exist: gender-typed behavior, for example, may be a condition of gender identity, but most individuals who are gender-atypical in behavior are not transgender (Berenbaum, 2018). Significantly, fuzzy-sets allow consideration of combinations of conditions that are sufficient or necessary for varying degrees of membership in a binary category (Pennings, 2003) and have advantages for understanding causality over conventional techniques such as logistic or linear regressions when, for example, different conditions yield the same outcome (Graham et al., 2019). Therefore, applying a fuzzy-set approach in future research may be prove useful in furthering our understanding of the complex determinants of gender identity and the role of forenames in gender development.

In sum, the present research provides the first evidence that the perception of forename gender typicality is associated with gender-linked behavior within groups of women and men. Although the results are consistent with the predictions of theories of forename effects on gender development (e.g., Pilcher, 2017), the study design and the homogeneous nature of our sample are limitations to be addressed in future research using more diverse populations and additional measures of gender-linked behavior, including measures of the multi-dimensional nature of gender (Egan and Perry, 2001). In addition, the retrospective nature of the PSAI may have limited our ability to document stronger effects of forenames on gender-linked social interests, suggesting that it may also be informative in future research to include eye-tracking measures of visual interest on a variety of gender-linked stimuli, including childhood toys and activities (e.g., Alexander and Charles, 2009). Despite these limitations, our novel findings suggesting forename typicality is a factor contributing to the established variability in gender role behavior clearly strengthen the call to renew research on the role of forenames in gender development (Robnett, 2017).

## Data Availability Statement

The raw data supporting the conclusions of this article will be made available by the authors, without undue reservation.

## Ethics Statement

The studies involving human participants were reviewed and approved by Texas A&M University Institutional Review Board. Written informed consent for participation was not required for this study in accordance with the national legislation and the institutional requirements.

## Author Contributions

GA conceived of the study hypothesis, contributed to the study design, completed the data analyses, and wrote the first draft of the manuscript. KJ contributed to the study hypothesis and study design, facilitated data collection, completed data processing, organized the database, created figures, and provided editorial feedback. JL and TH contributed to the study design and provided editorial feedback for the manuscript. All authors contributed to manuscript final revision, read, and approved the submitted version.

### Conflict of Interest

The authors declare that the research was conducted in the absence of any commercial or financial relationships that could be construed as a potential conflict of interest.
